# Lifestyle medicine in Parkinson’s disease

**DOI:** 10.1007/s00702-025-03059-y

**Published:** 2025-11-12

**Authors:** Patrick Süß, Martin Regensburger, Heiko Gassner, Jürgen Winkler

**Affiliations:** 1 Department of Molecular Neurology, University Hospital Erlangen, Friedrich-Alexander-Universität Erlangen-Nürnberg, Schwabachanlage 6, 91054 Erlangen, Germany; 2 Deutsches Zentrum Immuntherapie (DZI), University Hospital Erlangen, Erlangen, Germany; 3 Digital Health and Analytics, Fraunhofer Institute for Integrated Circuits IIS, Erlangen, Germany

**Keywords:** Parkinson’s disease, Lifestyle, Environment, Physical activity, Nutrition, Prevention

## Abstract

Lifestyle medicine is an evolving concept integrating preventive and therapeutic approaches related to physical activity, nutrition, sleep, stress management, social interaction, and cessation of substance abuse aiming to optimize health outcomes. Lifestyle consultation plays an essential and constantly growing role in clinical settings. In this review, we highlight the usability of lifestyle medicine as a framework for both well-established and novel roles of environmental triggers and non-pharmacological prevention and treatment in Parkinson’s Disease (PD). We summarize the current understanding of the modulation of PD risk and progression by lifestyle-related domains and provide a review of recent insights from approaches targeting physical activity and stress management as well as dietary interventions, including general dietary patterns, individual food groups, supplements, and microbiota-directed strategies in the context of PD. Importantly, we also identify current knowledge gaps and provide an outlook for future strategies to implement multidimensional, disease-specific, and stratified lifestyle medicine in the prevention and treatment of PD.

## Introduction

Parkinson’s disease (PD) is both the most frequent neurodegenerative movement disorder and the fastest growing neurological disease worldwide (Bloem et al. 2021). Most recent projections estimate an increase in the global prevalence of PD from 11.8 million cases in 2021 (Luo et al. 2024) to more than 25 million cases in 2050 (Su et al. 2025). This increase is mainly attributed to global ageing and underpins the high need for preventive approaches targeting lifestyle and environmental factors linked to PD, accumulating over lifetime.

In general, preventive measures can be differentiated into primary, secondary, and tertiary prevention, depending on their specific aims and targeted population (Ben-Shlomo et al. 2024). While primary prevention in PD aims to reduce disease incidence by general measures at the level of an entire population independent of individual risks for a distinct disorder, secondary prevention aims to the delay of the onset or the slowing of progression in patients at risk of developing manifest PD based on genetic predisposition or prodromal symptoms like hyposmia, constipation, or Rapid Eye Movement (REM)-sleep behavior disorder (RBD) (Ben-Shlomo et al. 2024; Postuma and Berg 2016). Of note, the potential of secondary prevention in PD is endorsed by (1) its slow progressive nature with preclinical and prodromal phases lasting for decades and (2) recent diagnostic advances in biomarker-based detection methods for premotor stages. In particular, seed-amplification assays may be able to detect spreading species of α-synuclein, the major protein accumulating in the brain and closely linked to initial neurodegenerative processes in PD patients (Bloem et al. 2021). While the analysis in patient-derived biospecimens still requires further diagnostic accuracy in order to achieve high sensitivity, specificity, and inter-laboratory validity (Bräuer et al. 2025; Kluge et al. 2022; Okuzumi et al. 2023; Shahnawaz et al. 2017), it already fueled efforts towards novel biomarker-based diagnostic frameworks for PD (Höglinger et al. 2024; Simuni et al. 2024). Finally, tertiary or symptomatic prevention aims to slow the further progression and prevent future adverse symptoms in patients with motor-manifest PD (Ben-Shlomo et al. 2024).

In the present review, we aim to give an oversight of the current knowledge regarding lifestyle-related modifiable factors increasing or reducing the risk of developing and/or modifying the disease course of PD. The communication of established and emerging modifiable risk factors of PD is important at all three levels of prevention, including the broad public awareness among the general population for primary prevention and the design of longitudinal studies for secondary prevention measures in patients at risk. Lifestyle adaption in clinical settings mainly refers to tertiary prevention and requires longitudinal follow-up consultations, the incorporation of patients’ caregivers, families, and living environments as well as an interdisciplinary concept involving physiotherapists, nutritional therapists, and others.

The term “lifestyle” was inaugurated by the Austrian physician and psychotherapist Alfred Adler to define types of individual personalities and social interaction patterns rooted in early childhood (Adler 1929). In its current use, “lifestyle” refers to an individual’s set of behavioral choices in a defined socioeconomic context. Lifestyle is closely interconnected with an individual’s social, occupational and natural living environment, which determines the degree of exposure to environmental factors further modifying disease risk and its progression. In the context of PD, such environmental or occupational risk factors are well established and include pesticides like paraquat and organochlorides (Ascherio et al. 2006; Ben-Shlomo et al. 2024), air pollution (Murata et al. 2022), particulate matter (Krzyzanowski et al. 2023; Zhang et al. 2025), organic solvents like trichloroethylene (Goldman et al. 2012), and potentially also more recently identified factors like microplastic (Erro et al. 2025).

In the last two decades, the term “lifestyle medicine” has arisen, aiming to improve health outcomes by focusing on physical exercise, nutrition, sleep, stress management, social interactions, and cessation of substance use (Lippman et al. 2024). We will describe the current evidence for the potential of these different domains to decrease the risk of developing PD and/or to modify the disease course of PD. Moreover, we will highlight interventional approaches related to lifestyle medicine in PD, point out current knowledge gaps, and try to provide an outlook on future developments.

## Methods

We conducted a narrative (non-systematic) review focused on lifestyle medicine in PD. PubMed/MEDLINE was searched for articles published 2010-2025 using combinations of the terms “lifestyle”, “prevention”, “physical exercise”, “physiotherapy”, “nutrition”, “Mediterraean Diet”, “coffee”, “milk”, “nutraceuticals”, “prebiotics”, probiotics”, “sleep”, “stress”, “social interaction”, “substance use”, “smoking” and “alcohol” with “Parkinson’s Disease”. Reference lists of included papers were screened to identify additional relevant studies. To provide further context where considered relevant, selected articles published prior 2010 were also implemented. Given the breadth of the topic, recent high-quality evidence syntheses (reviews/meta-analyses) and key primary studies most relevant to the review questions were prioritized. No formal risk-of-bias assessment was performed. The last PubMed search was performed on September 24, 2025.

## Physical activity in PD

Among lifestyle-associated factors, physical exercise is broadly consented to reduce the risk of developing PD as a primary preventive measure. Although class I evidence for a risk- or disease-modifying effect is still missing, different studies indicate strong and meaningful effects of physical exercise. A prospective study of more than 200,000 American participants concluded that moderate to vigorous physical exercise between the age of 35-39 years or in the decade prior to the study was associated with a reduced risk of developing PD in the following years, whereas light physical activity showed no effect (Xu et al. 2010). More recently, a potential reduction of PD risk was restricted to high-frequency and intense physical exercise in a large British patient cohort (Veronese et al. 2024). Of note, studies often lack quantitative and metric measures for physical activity. A meta-analysis estimating physical activity by metabolic equivalent of task (MET)-hours per week reported a continuous decline of PD risk particularly in men, with a risk reduction by 17% for an increase of moderate to vigorous physical activity by 10 MET hours per week, e.g. corresponding to brisk walking for ca. 3 h per week (Fang et al. 2018). Besides the potentially primary preventive nature of physical exercise, its tertiary preventive effects on the progression of PD were intensively studied, particularly in early and intermediate disease stages. High-intensity treadmill exercise over 6 months was associated with a stabilization of motor symptoms, whereas moderate exercise was not shown to prevent a further decline of motor functions (Schenkman et al. 2018), again indicating a dose-dependent effect for physical exercise. This was also observed in a time-to-event analysis showing that intensive physical activity measured by MET hours prior to disease onset was linked to a delayed time interval until reaching milestones of motor progression in PD patients (Hu et al. 2024). A retrospective cohort study on patients with early PD reported that maintained physical activity over time slowed the deterioration of postural and gait stability (Tsukita et al. 2022). The importance of continuous physiotherapy in PD patients was evident during the COVID-19 pandemic, when clinical deterioration of PD patients was strongly attributed to reduced physical activity and very limited access to physiotherapy (Ineichen et al. 2021). Importantly, the deficits were not compensated by later resumption of physiotherapy (Ineichen et al. 2023). There is a broad consensus on the recommendation of physical exercise and physiotherapy in PD, which should ideally be embedded in a multidisciplinary rehabilitative care approach including occpupational therapy, speech/language therapy and neuropsychology (Goldman et al. 2024). However, it is still unclear if there is an ideal or specific type of exercise or physical therapy for PD patients. Current physiotherapy guidelines for PD patients provided by the American Physical Therapy Association include a range of different therapies including balance training, gait training, resistance training, task-specific training, aerobic exercise, and external cueing (Osborne et al. 2022). A recent Cochrane review summarized the degree and confidence of currently provided evidence for effects of different physical exercise interventions on motor symptoms and quality of life in PD patients obtained by randomized-controlled trials. Most types of interventions included had beneficial effects compared to passive control groups (Ernst et al. 2024). The most pronounced effects on motor symptoms in PD were reported for dance and gait/balance/functional training. Furthermore, effects on quality of life were most profound for aqua-based training, however no significant differences were concluded between intervention types. This observation is most likely due to the overall moderate effect sizes and limited confidence for the evidence provided by most studies based on low numbers of study participants. Moreover, head-to-head comparisons between different types of physical exercise are lacking (Ebersbach 2025; Ernst et al. 2024). The provided evidence for the safety of physical interventions in PD was very limited, with most interventions overall appearing safe (Ernst et al. 2024). Further limitations of current studies are the strong focus on early to moderate disease stages and the lack of information on single, well-defined motor symptoms which could be a particular target of specific interventions, e.g. gait impairment (Ebersbach 2025; Ernst et al. 2024). Moreover, the secondary preventive potential of physical exercise in prodromal PD cohorts has not been investigated.

Mechanisms underlying potentially beneficial effects of physical exercise on PD risk and progression are insufficiently understood. One potential mode of action may be the restoration of mitochondrial dysfunction, which is considered a major pathophysiological driver of PD (Schon et al. 2025). This is supported by the elevation of the myokine irisin in the serum of PD patients after 12 weeks of regular exercise (Zhang et al. 2023), which positively correlated with improved balance function and provided indirect protection of mitochondria and neuroprotection in toxin-induced models of PD (Zhang et al. 2023). Moreover, physical exercise may ameliorate PD via neurotrophic factors. Brain-derived neurotrophic factor (BDNF) was induced in the serum of PD patients by intense rehabilitation therapy including aerobic exercise, along with improved motor function (Frazzitta et al. 2014). Further potential mechanisms underlying the assumed effects of physical exercise on PD include immunomodulation, alterations of neural network activity, an increase of cerebral perfusion and glymphatic flux as well as an influence on gut microbiota (Wilson et al. 2025). These and other mechanisms require further investigation by future studies combining exercise interventions with analyses of fluid, imaging or electrophysiological biomarkers.

To conclude, physical exercise may reduce the risk to develop PD and should be recommended to PD patients as there is an evidence-based, disease modifying benefit, while the particular type of exercise may be less relevant. Given the dose-dependence and the relevance of long-term continuation of physiotherapy, adherence to physiotherapy is essential, but neglected by the majority of studies (Allen et al. 2012). Future interventions should analyze adherence to physical exercise and implement strategies to overcome barriers for physical exercise in PD patients and improve adherence, e.g. by structured supervision and coaching, activity monitoring, goal setting, and positive feedback (Ahern et al. 2024; Ellis et al. 2013). In the future, interventions specifically tailored towards PD may be particularly effective and the specialization of therapists for the care of PD patients within integrated care networks may result in reduced PD-related complications and mortality as well as lower health costs (Ypinga et al. 2018).

An exception to the general recommendation of physical exercise to prevent PD may be related to specific contact sports with an increased risk and frequency of head injury. A history of participating in American football was recently linked to higher odds of developing PD, especially with long duration or high professional sport level (Bruce et al. 2023). While increased mortality of neurodegenerative diseases including PD was reported in Scottish former professional soccer players (Mackay et al. 2019), a swedish study of former professional soccer players found an increased frequency of Alzheimer’s disease (AD), but a reduced frequency of PD (Ueda et al. 2023). The focus on head injury-prone sports is based on previous studies proposing repeated traumatic brain injury (TBI) as a risk factor for PD. Adjusted hazard ratios for PD risk were reported to correlate with TBI severity (Gardner et al. 2015) and increased PD risk by TBI was also reported for head injuries early in life, long before the diagnosis of PD was made (Taylor et al. 2016). However, the impact of TBI on PD risk remains controversial. A recent case-control study was not able to confirm an association of TBI in general nor a correlation between frequency, severity, or time of TBI with PD and other synucleinopathies (Hasan et al. 2020).

## Nutrition in PD

Nutrition in PD tremendously raises a growing attention due to the early and prominent presence of gastrointestinal symptoms in PD patients (Sampson et al. 2025). In terms of PD pathogenesis, Braak’s hypothesis significantly contributed to this notion based on findings that distinct α-synuclein species originate in the gut and may propagate rostrally to the brain (Braak et al. 2003; Schmitt et al. 2023; Shannon et al. 2012). Moreover, gut microbial composition is altered in PD patients and implicated in PD pathogenesis (Munoz-Pinto et al. 2024; Romano et al. 2025; Wallen et al. 2022). These findings led to the development of multiple nutritional probiotic, and other microbiota-targeting studies to modulate early pathophysiological events in PD (Hamilton et al. 2024; van der Berg et al. 2024). Conversely, certain types of nutrition may also have negative effects on developing PD and/or modifying the disease course. In the following sections, we will highlight the current understanding of the role of nutrition in PD ranging from general nutritional indices and patterns to individual foods and supplements, before we will summarize microbiota-targeted nutritional interventions in PD.

### Nutritional indices and patterns

Several indices have been developed to assess nutritional quality and its impact on health outcomes. Among those, the Alternate Healthy Eating Index (AHEI) and the Alternate Mediterranean Diet Score (AMED) follow general dietary recommendations to reduce chronic disease risk or local cuisine in the Mediterranean area, respectively, and are based on diets rich in vegetables, fruit, fish and cereals with low intake of red meat and saturated fat. Of note, higher AHEI and AMED counts were associated with a reduced risk to develop PD (Gao et al. 2007) or PD-related prodromal symptoms (Molsberry et al. 2020). In line with this, Mediterranean diets were reported to reduce the risk of manifest PD (Yin et al. 2021) as well as PD-related prodromal symptoms (Maraki et al. 2019). A more specific diet tailored towards neurodegenerative diseases, the Mediterranean-Dietary Approaches to Stop Hypertension (DASH) Intervention for Neurodegenerative Delay (MIND) diet, which includes leafy greens, beans, and berries and minimizes cheese, butter, and margarine consumption (Tosefsky et al. 2024), even further reduced PD risk compared to Mediterranean diets and might even slow the progression of parkinsonism in elderly (Agarwal et al. 2018). Other dietary patterns including plant-based and ketogenic diets showed first hints towards reducing PD risk in smaller studies, but require further confirmation (Tosefsky et al. 2024).

As anti-inflammatory effects are considered a major protective mechanism behind the upper-mentioned dietary patterns (Tosefsky et al. 2024), the more mechanistically driven dietary inflammatory index (DII) has been developed, which integrates the current evidence of dietary induction of pro- and anti-inflammatory factors (Hebert et al. 2019). As such, the DII was applied to link a broad variety of chronic diseases with pro-inflammatory nutritional patterns (Marx et al. 2021). Recently, higher DII values were suggesting to increase the risk of developing prodromal PD symptoms (Balomenos et al. 2022), which is in line with the presumed pathogenic contribution of inflammatory mediators to PD (Tansey et al. 2022).

Despite the evidence for beneficial effects of healthy diet patterns especially on primary PD prevention, a recent study reported that PD patients exhibit a lower dietary quality compared to controls based on the Healthy Eating Index (HEI)-2015 (Kwon et al. 2023), which illustrates the high importance of dietary consultation and recommendations in PD patients. Lower nutritional quality of PD patients may be partially attributed to disease- and treatment related factors including constipation, hyposmia, and impulsive-compulsive behavior induced by dopamine agonist treatment (Kwon et al. 2023).

A challenge to the broadly accepted paradigm that healthy diet is beneficial in the context of PD risk is the longstanding and conflicting hypothesis that high levels of cholesterol and urate, which tend to be lower in people with high HEI-2015 scores (Faraji et al. 2024; Nie et al. 2022), were protective against PD (Alonso et al. 2007; Gao et al. 2016; Simon et al. 2007) and slowed PD progression (Huang et al. 2011; Schwarzschild et al. 2008). However, more recent studies point to a reverse causation and suggest that lower cholesterol and urate levels are caused by prodromal PD itself (Seifar et al. 2022; Wang et al. 2021). In the context of urate, a Mendelian Randomization study did not detect an effect on PD risk (Kia et al. 2018) and a randomized clinical trial with inosine to increase urate levels failed to meet clinical endpoints in PD patients (Schwarzschild et al. 2021). Collectively, general nutritional recommendations including Mediterranean diet and adherence to the HEI-2015 may reduce PD risk as a primary preventive measure and may also be applicable to prodromal and manifest PD patients for secondary and tertiary prevention, but a future task is to develop a disease-specific nutritional index or dietary pattern to maximize the potential benefits of diets in PD.

### Food groups

In addition to dietary patterns and indices, foods are categorized based on their preparation and composition. The Nova food classification system assigns foods to one out of four groups based on the degree of industrial processing, ranging from unprocessed foods (Nova 1) to ultra-processed foods (Nova 4) (Martinez-Steele et al. 2023). An increased consumption of ultra-processed food was related to multiple adverse outcomes (Lane et al. 2024). Recently, a longitudinal follow-up study revealed that long-term intake of ultra-processed food increased the risk of prodromal non-motor PD-related symptoms including RBD, depression, and constipation (Wang et al. 2025). In line with this, foods associated with lower PD progression based on patient-reported outcomes (PROMs) included fresh fruit, vegetables, and herbs (Nova 1), while canned fruit and vegetables (Nova 3) as well as fried foods (Nova 3–4) were linked to more rapid disease progression (Martinez-Steele et al. 2023; Mischley et al. 2017).

As a part of their lower-quality nutrition, PD patients were reported to consume increased amounts of carbohydrates (Kwon et al. 2023), which was confirmed in a second study and linked to lower quality of life, increased constipation and an elevation of required levodopa equivalent daily dose (LEDD) (Palavra et al. 2021). This supports a bidirectional link between PD and diabetes reflected by a diet-related pre-existing comorbidity and its link to increased PD risk (Azami et al. 2024), progression of PD (Athauda et al. 2022), and PD-related cognitive impairment (Bohnen et al. 2014). Thus, the limitation of carbohydrate consumption and management of diabetes may contribute to primary, secondary, and tertiary prevention in PD. Underlying mechanisms may include impaired insulin-dependent function and survival of dopaminergic neurons, inflammatory pathways, mitochondrial dysfunction (Ruiz-Pozo et al. 2023), and glycation of α-synuclein (Vicente Miranda et al. 2017).

Furthermore, the influence of dairy consumption on PD risk and progression was thoroughly investigated, but remains controversial. A meta-analysis reported a slight increase in PD risk by high levels of milk and cheese intake in men (Jiang et al. 2014). In a cohort of men from Hawaii, high milk intake between the years 1965–1968 was associated with neuronal loss in the substantia nigra as detected by brain autopsy between 1992 and 2004, but these alterations were strongly related to a contamination of milk with pesticides at that time (Abbott et al. 2016). An analysis of two large prospective cohorts concluded gender-independent elevation of PD risk mainly upon intake of low-fat dairy intake (Hughes et al. 2017). Moreover, an estimation of disease course based on PROMs suggested accelerated PD progression related to cheese, cream, and yoghurt, but not milk and butter intake (Mischley et al. 2017). In contrast to these findings, a Japanese case-control study did not confirm a link between dairy product consumption and PD risk (Miyake et al. 2011). More recently, the role of dairy products in PD were addressed by Mendelian Randomization studies. This approach is increasingly being applied in the research on risk factors for neurodegeneration, as the association of genetic variants predisposing for exposure to a potential risk factor, e.g. dairy intake, with PD risk is less vulnerable to reverse causation and other confounders. A Mendelian Randomization study in 2022 found that a variant in the lactase gene predicting higher dairy intake was linked to an increased risk of PD patients in males (Domenighetti et al. 2022). Further studies are necessary to reveal the role for specific dairy products in the context of primary, secondary, or tertiary PD prevention in relation to specific microbial signatures, ethnic backgrounds, and disease trajectories.

### Individual foods, beverages, and supplements

One of the best-studied individual dietary components in the context of PD is coffee. Regular consumption of coffee and other sources of caffeine was reported to reduce PD risk (Ascherio et al. 2001) and linked to a slower progression of PD (Paul et al. 2019). This was corroborated by a recent meta-analysis (Hong et al. 2020). Of note, PD risk reduction by coffee was observed to be more pronounced in men and dose-dependent until reaching a maximum at 3 cups of coffee intake per day (Qi and Li 2014). Underlying mechanisms discussed include effects on the composition of gut microbiota, but also by neuronal effects by blocking striatal adenosin A_2A_ receptors, which can already be observed after one cup of coffee (Ishibashi et al. 2022). In line with this, the approved A_2A_ receptor antagonist istradefylline reduces OFF-symptoms in PD patients (Hauser et al. 2021). However, a clinical trial on caffeine treatment in PD patients showed increased dyskinesia, but failed to induce motor improvements in PD patients within 6 months (Postuma et al. 2017). In summary, coffee may contribute to primary and tertiary PD prevention, but the underlying mechanisms induced by caffeine and potentially by other components of coffee still need to be fully elucidated.

Besides caffeine, the effect of several other individual foods or ingredients on PD risk and progression were examined. Results largely overlap with the above-mentioned patterns of beneficial effects by Mediterranean diets and unprocessed foods. PD progression based on PROMs was observed to be slowed by fresh fruit, fresh vegetables, nuts, seeds, fish, olive oil, coconut oil, herbs, and spices, while more rapid progression was associated with consumption of fried food, red meat, and diet soda (Mischley et al. 2017).

The term ‘nutraceuticals’ refers to dietary supplements applied for medical prevention and treatment. In the context of PD, several nutraceuticals were proposed based on putative anti-oxidative, anti-inflammatory or other modes of action (Sharma et al. 2025), but only few showed beneficial effects in PD patients. Fish oil containing high amounts of omega-3 polyunsaturated fatty acids were associated with a decreased PD risk (Lin et al. 2023) and slower PD progression (Mischley et al. 2017). Combined supplementation of omega-3 and omega-6 polyunsaturated fatty acids in conjunction with vitamin A, vitamin E, and γ-tocopherol for 30 months was linked to reduced motor progression in a small, randomized, placebo-controlled trial (Pantzaris et al. 2021). Among other supplements included in the aforementioned study assessing PD progression based on PROMs, only coenzyme Q10 showed a significant benefit (Mischley et al. 2017), but previously failed to meet clinical endpoints at high dosages in a study of patients with early PD (Beal et al. 2014). Despite these negative data, supplementation of coenzyme Q10 as an inherent component of the mitochondrial respiratory chain may by particularly beneficial in PD subtypes more strongly related to mitochondrial dysfunction, including monogenic PD cases linked to the genes *PINK1*, *PRKN*, and *DJ-1* (Lin and Beal 2006; Schon et al. 2025), which needs to be further studied in stratified PD subcohorts. Vitamin D deficiency was associated with increased PD risk as well as motor and non-motor symptoms in PD, but so far showed conflicting results in clinical trials (Barichella et al. 2022). Recently, supplementation of the modified amino acid acetyl-DL-leucin has gained larger attention based on a case report of two patients with prodromal signs of PD including RBD treated with acetyl-DL-leucin over 18 and 22 months, respectively. Strikingly, a reversal of the loss of the striatal dopamine-transporter single-photon emission computerized tomography (DAT-SPECT) signal and the PD-associated imaging patterns in ^18^F-fluorodeoxyglucose positron emission tomography (FDG-PET) was reported, potentially indicating a disease-modifying effect (Oertel et al. 2024). However, future controlled studies in larger cohorts are crucial to test this rather anecdotal report, i.e. by excluding acetyl-DL-leucin mediated alterations in DAT tracer binding. In contrast to the potentially beneficial supplements mentioned above, iron supplementation was suggested to accelerate PD progression (Mischley et al. 2017). Overall, the current evidence for nutriceuticals in PD is very limited. Future controlled studies for individual supplements and their combination in different disease stages are urgently needed.

### Microbiota-targeted therapies

Although several of the above-mentioned diets, foods, or supplements may have the potential to modify risk and disease progression of PD by modulating gut microbiota, specific nutritional interventions directly targeting gut dysbiosis in PD are of major interest. Given the fact that PD-related microbial alterations may already be present in early diseases stages, and patients with prodromal symptoms like RBD (Huang et al. 2023; Palacios et al. 2023), microbiota-targeting interventions harbor primary, secondary, and tertiary preventive potential.

First, microbial modulation may be achieved by prebiotic diets, which provide metabolic substrates to microbiota and promote the growth of potentially “protective” strains and/or its beneficial metabolites. A key dietary component of prebiotic treatments are dietary fibers, which are discussed as key mediators of the beneficial effect conveyed by a Mediterranean diet. Distinct bacterial strains are able to metabolize dietary fibers into short-chain fatty acids (SCFA), including acetate, propionate, butyrate, iso-butyrate, valerate, and iso-valerate (Bedarf et al. 2025). Levels of different SCFA were reduced in the feces and increased in the serum of PD patients, correlating with microbial changes and disease severity (Chen et al. 2022). A dietary intervention with resistant starch as the only fiber source already reported an improvement of depressive and other non-motor symptoms in PD patients after a trial period of 8 weeks, yet without causing significant changes in the gut microbiome (Becker et al. 2022). Interestingly, a more diverse supplementation of dietary fibers by a bar containing resistant starch, rice brain, resistant maltodextrin, and inulin was found to partially restore microbial changes and altered SCFA levels after just 10 days (Hall et al. 2023). These changes were accompanied by a reduction in gastrointestinal symptoms as well as a slight, but significant reduction in neurofilament light-chain levels, raising the possibility of a neuroprotective effect by dietary fibers in PD (Hall et al. 2023). A very recent intervention study evaluated a prebiotic fiber-based diet combined with lactulose in PD patients and household controls over a period of 4 weeks. Interestingly, prebiotic treatment did not alter PD-related dysbiosis, but partially reversed dysbiosis-related metabolic changes, including an increase in fecal SCFAs, but also other metabolites implicated in gut-brain communication (Bedarf et al. 2025). In summary, current studies on prebiotic interventions showed promising results, but further studies with larger sample sizes and longer treatment durations are required to reveal clinical meaningful effects on the microbial dysequilibrium and the course of PD. Moreover, mechanisms linking gut microbiota and their metabolites to PD pathogenesis are still insufficiently understood. For example, SCFAs were proposed to modulate PD pathology via direct effects on neurons and the regulation of microglia-mediated neuroinflammation, but preclinical studies on the effects of SCFAs in PD mouse models provided conflicting results (Schmitt et al. 2023).

Besides prebiotic approaches, direct probiotic supplementation of beneficial bacterial strains is currently investigated as another treatment strategy. The growing number of small studies published so far focused on supplementation of different *Lactobacillus* or *Bifidobacterium* species (Hamilton et al. 2024). Of note, both taxa are already enriched in the stool of PD patients, as confirmed by a recent large meta-analysis (Romano et al. 2025). Nevertheless, positive effects of probiotic interventions were reported in all individual studies and confirmed in a meta-analysis regarding both motor and non-motor symptoms, specifically depression (Park et al. 2023). In contrast, evidence of probiotics on the modulation of PD-related dysbiosis are limited. Besides the substitution of different bacterial species, future studies also require larger cohorts and longterm trials to assess the potential of probiotic treatments in PD.

While prebiotic and probiotic approaches aim to modulate microbiota to reduce the risk and progression of PD, microbiota may also be targeted to improve treatment response to levodopa. Of note, levodopa was shown to be metabolized by different bacteria including *Enterococcus* species, thereby decreasing the bioavailability and therapeutic effect for patients (Maini Rekdal et al. 2019; van Kessel et al. 2019). This might be reverted by supplementation of SCFA conjugates of honokiol, a polyphenol naturally occurring in the bark of Magnolia trees recently found to inhibit bacterial degradation of levodopa (Cheng et al. 2025).

Taken together, nutrition may be a relevant lifestyle-related modifiable factor influencing the risk of PD. Dietary interventions, particularly targeting gut microbiota by prebiotic or probiotic appoaches, have mainly been studied in the context of primary prevention, while secondary and tertiary nutritional prevention in PD, which might include substantially different strategies, are less well examined. Overall, nutrition-targeted lifestyle medicine holds great potential in PD. To exploit this potential, future studies should combine probiotic and prebiotic treatments, which has been rarely performed so far (Barichella et al. 2016; Ibrahim et al. 2020). As pathophysiological insights are increasing and dietary, pre-, and probiotic intervention protocols are becoming more sophisticated, future approaches may more specifically target individual metabolites or bacterial strains related to PD. Alternatively, considering the high variability of microbial signatures between individual PD patients, personalized lifestyle medicine might one day be able to provide tailored dietary, prebiotic, and probiotic recommendations for each individual patient.

## Sleep in PD

Sleep disorders represent an instructive example of the challenges when aiming to distinguish effects on PD risk and progression from reverse causation. Sleep disturbances are well accepted symptoms of prodromal PD, especially due to the high specificity of RBD for synucleinopathies (Iranzo et al. 2024). Besides RBD, Restless-Legs-Syndrome (RLS) (Wong et al. 2014) as well as insomnia (Hsiao et al. 2017; Schrag et al. 2015) were related to prodromal PD. Regarding the manifest PD population, insomnia is reported in > 50%, followed by RBD, excessive daytime sleepiness and RLS-related sleep disturbance (Dodet et al. 2024). The number of sleep disorders increases with disease duration (Dodet et al. 2024). Obstructive Sleep Apnea (OSA) was associated with increased risk of PD and more severe motor impairment (Maggi et al. 2024), but might as well be linked to prodromal PD. Daytime sleepiness was related to an increased PD risk after a median follow-up of almost 12 years (Chen et al. 2023) and linked to more severe non-motor and motor symptoms (Maggi et al. 2023), but could also be another feature of prodromal PD. Moreover, daytime sleepiness can be caused by comorbid OSA or treatment with dopamine agonists in manifest PD patients (Anderson et al. 2025). These differential physiological underpinnings point towards different mechanisms: RBD may reflect early brainstem pathology in the disease process (Boeve et al. 2007). Moreover, no lifestyle interventions are known to affect the manifestation and/or symptom severity of RBD. Importantly, a prospective study showed that a reduction in sleep quality and sleep duration correlated with PD incidence within 2 years, but this correlation diminished with longer follow-up periods, further supporting the notion that sleep disturbance is rather a feature of prodromal PD than an independent PD risk factor (Lysen et al. 2019). Although the treatment of comorbid sleep disorders in PD is recommended and likely to have a beneficial (indirect) influence on PD symptoms as well (Anderson et al. 2025), further studies are required to clarify the bidirectional link between PD and sleep disturbances.

In contrast to sleep disturbances, a high quality of sleep was correlated to a reduced PD risk after a follow-up period of 12 years (Veronese et al. 2024). Sleep quality includes multiple parameters like sleep duration, efficiency, latency, and time awake after sleep onset (Nelson et al. 2022), but precise lifestyle interventions improving sleep quality are understudied. While long-term adherence to the general recommendation of a sleep duration of 7 h per night may reduce overall morbidity and mortality (Watson et al. 2015), specific sleep-related lifestyle modifications for primary, secondary, or tertiary prevention of PD are not known.

## Stress management in PD

Stress is increasingly being acknowledged as a potential driver of PD. The number of previous stressful life events was observed to be positively correlated with PD in retrospective case-control studies (Rod et al. 2010; Vlajinac et al. 2013). Moreover, stressful life events may even directly provoke the onset of tremor in PD, making it difficult to differentiate from functional tremor (van der Heide et al. 2024b). While these observations indicate a potential role of stress management in primary and secondary PD prevention, stress may also aggravate symptoms in manifest PD patients and display a target for tertiary prevention. Among the prototypical motor symptoms in PD, tremor may be specifically worsened (Dirkx et al. 2020) and become less responsive to levodopa treatment (Zach et al. 2017) upon stress induced by cognitive load. Besides tremor, an online survey of 5000 PD patients also revealed stress-induced worsening of sleep problems, depression, bradykinesia, gait, and dyskinesia (van der Heide et al. 2021b). Mindfulness-based interventions are well-established therapeutic approaches to promote coping and reduce stress. Their principle relies on the non-judgmental concentration on the present moment (van der Heide et al. 2021a). In the last decade, several mindfulness-based intervention studies, which were partially combined with yoga (Kwok et al. 2019) or cognitive rehabilitation (Reitano et al. 2023), have reported short-term benefits on depression, anxiety, cognition, and quality of life in smaller PD patient cohorts (Kwok et al. 2019, 2023; Reitano et al. 2023; van der Heide et al. 2021a). Motor effects of mindfulness-based interventions in PD are less well studied, but were also reported to be beneficial (Kwok et al. 2019; Pickut et al. 2015). Recent developments include longer follow-up periods to investigate long-term effects (van der Heide et al. 2024a) as well as other stress-reducing interventions like compassionate mind training (Della Morte et al. 2024). In summary, stress management is an important factor, which can be implemented in lifestyle consultations of PD patients.

## Social interaction in PD

Social interaction is essential for emotional well-being. More importantly, loneliness was identified as an independent risk factor for mortality in the general population in a meta-analysis of 90 prospective cohorts including more than 2.2 million individuals (Wang et al. 2023). In the context of PD, loneliness was specifically linked to an increased risk of developing PD even after correction for multiple covariates including genetic risk and depression (Terracciano et al. 2023). Interestingly, this was not observed within the first five years of loneliness, which makes a reverse causation less likely (Terracciano et al. 2023). Besides PD risk, loneliness was proposed as a major driver of accelerated PD progression during the COVID-19 pandemic (Ineichen et al. 2021).

Furthermore, loneliness is highly prevalent in PD patients and more dependent on subjective functional impairment, social network size, and depression than the level of objective motor impairment (Gonzalez et al. 2025). In turn, loneliness confers functional impairment in PD (Gonzalez et al. 2025) creating a long-term vicious cycle in affected patients. Lifestyle interventions promoting social interactions therefore require further investigation. Of note, these interventions need to overcome hurdles for social interaction in PD patients, not only based on motor and speech impairment, but also including deficits in social cognition (Siripurapu et al. 2023), an impaired theory of mind (Trompeta et al. 2021), and an increased frequency of alexithymia (Fernandez-Fernandez et al. 2025). Recently, a prospective online cohort study was established in UK to investigate the impact of web-based social interaction on the risk of PD (Li et al. 2024).

Besides general social interaction and loneliness, family status is a major aspect of social lifestyle. Being married was related to higher total brain and hippocampal volumes (Grasset et al. 2025), but surprisingly proposed to increase the risk for dementia after 18 years (Karakose et al. 2025). The role of marital status in PD requires further prospective investigation. In female PD patients, a higher number of children was linked to a later age of disease onset, which may indicate a risk-reducing effect of childbearing potentially mediated by estrogen (Frentzel et al. 2017). A future integration of these and other social factors might enable a more thorough stratification of PD risk. Moreover, underlying reasons and mechanisms for the prospective effect of social lifestyle aspects on PD risk and progression need to be further systematically elucidated.

## Substance use in PD

Lastly, cessation of substance use is an essential element of lifestyle medicine. In particular, cessation of smoking can strongly reduce cardiovascular, respiratory, and cancer-related mortality, slowly approaching the mortality of non-smokers over approximately 20 years (Thomson and Islami 2024). In the context of neurodegenerative diseases, smoking cessation, but not reduction, reduced the risk to develop dementia. However, there is a longstanding notion in the field that smoking may reduce the risk of PD. An early study on twin pairs found an inverse correlation between the dose of smoking and PD risk, particularly in monozygous twins (Tanner et al. 2002). This was more recently supported in large follow-up studies on British cohorts (Mappin-Kasirer et al. 2020; Veronese et al. 2024). A 65-year follow-up in a cohort of British doctors indicated a dose-dependent association between smoking and PD risk, with the lowest risk present in continuing smokers (Mappin-Kasirer et al. 2020). The therapeutic potential behind this observation was explored in a clinical trial on transdermal nicotine application in early PD patients, which failed to meet clinical endpoints (Oertel et al. 2023). This raises the possibility that components other than nicotine might confer protective effects in PD or that that the observed link between smoking and reduced PD risk is due to reverse causation. In line with this, a case-control study by Ritz et al. found that smoking was less common among PD patients and smoking cessation was easier for them compared to controls (Ritz et al. 2014). The authors argue that previous data linking smoking to a reduced risk of PD may rather be explained by a reduced addictive potential of smoking representing a premotor PD feature (Ritz et al. 2014). Moreover, among manifest PD patients, smoking was related to a faster cognitive decline (Paul et al. 2019). Overall, there is still controversy about the effects of smoking on PD risk and/or progression. Given the negative effects of smoking on overall health, it should not be part of lifestyle recommendations in PD patients or risk carriers.

Similar to smoking, the role of alcohol drinking in PD risk and/or progression remains controversial (Reichmann et al. 2022). While heavy drinking (Paul et al. 2019) or previous hospitalization related to alcohol abuse (Eriksson et al. 2013) was linked to a highly elevated PD risk, reports on the effects of lower amounts of alcohol or individual alcoholic beverages are conflicting (Reichmann et al. 2022). While individual studies report a reduced risk of PD with low-amount intake of beer (Liu et al. 2013) and a reduced disease progression rate with intake of wine (Mischley et al. 2017), large prospective cohort studies showed no overall association between alocohol consumption and PD risk independent of the beverage type (Palacios et al. 2012; Peters et al. 2020). Overall, the current knowledge on the role of alcohol consumption in PD risk and/or progression, does – beyond general health-related considerations – not allow PD-specific recommendations.

### Conclusion and outlook

**Fig. 1:**
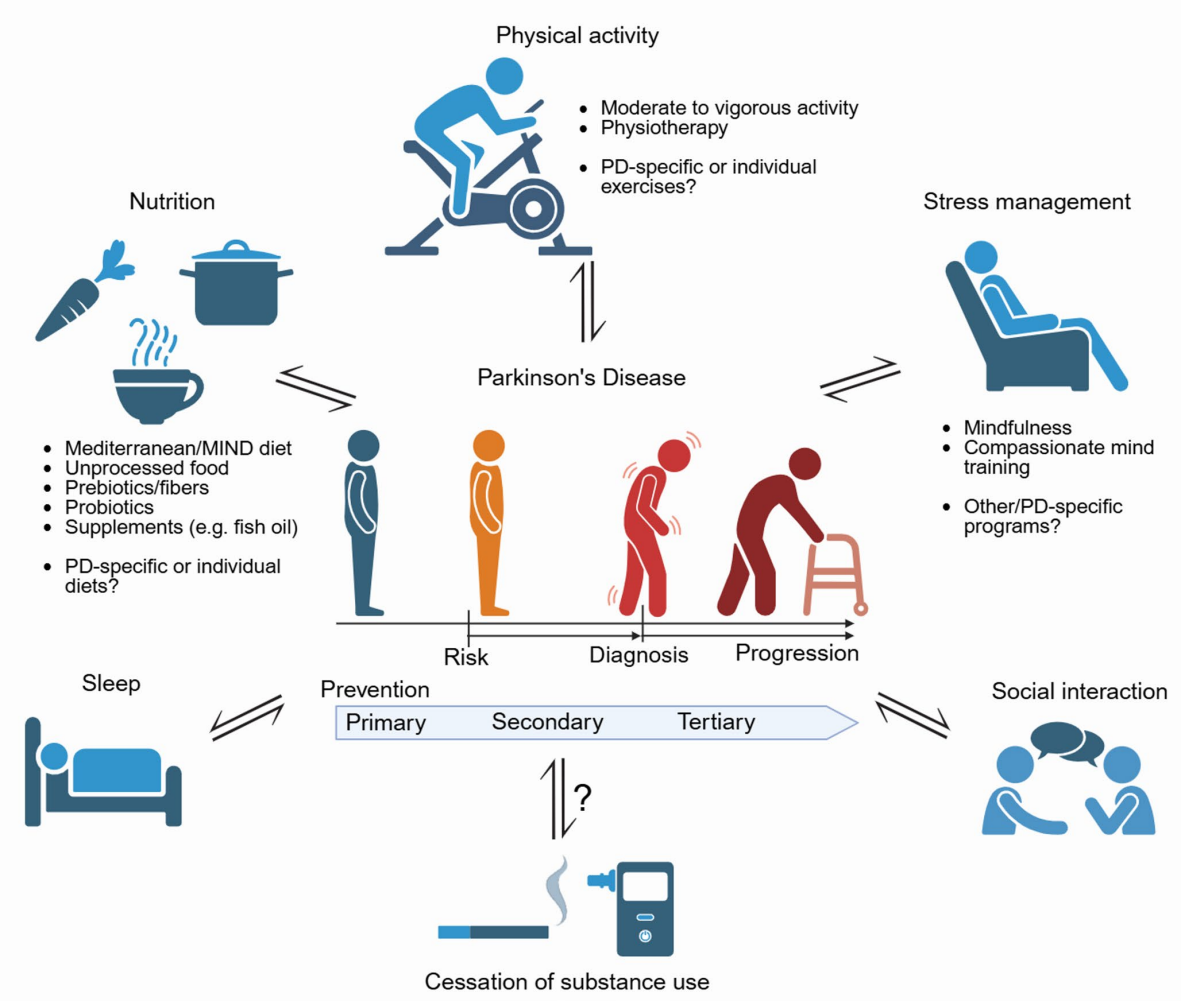
Lifestyle medicine in Parkinson’s Disease. The six domains of lifestyle medicine, physical activity, nutrition, sleep, stress management, social interaction, and substance abuse reciprocally interact with Parkinson’s Disease (PD). They can influence the risk and progression of PD, but are also modulated by PD itself, potentially also during prodromal disease stages. Lifestyle interventions for the prevention or treatment of PD have been studied regarding nutrition, physical activity, and stress management. Diet-related recommendations include a Mediterranean-like dietary pattern and unprocessed or low-processed food. Prebiotics and probiotics showed beneficial effects and are under continuing intensive investigation, while evidence for benefits of supplements is limited, with fish oil being among very few promising candidates. Physical activity exerts positive effects in a dose-dependent manner, and continuous physiotherapy is key to slow PD progression. Mindfulness was repeatedly reported to reduce the risk and progression of PD, while compassionate mind training and other strategies require further investigation. Lifestyle-related recommendations can be applied at different stages. Primary preventive measures aim to reduce PD incidence of the general population (dark blue). Secondary prevention can be applied to delay the progression of persons at risk (orange) towards disease onset (red). Tertiary prevention aims to slow down disease progression (dark red). Overall, recommendations for lifestyle medicine in PD are so far rather general. Integrative approaches covering several lifestyle medicine-related domains as well as more PD-specific interventions hold great preventive and therapeutic potential for the future. Created in BioRender. Süß, P. (2025) https://BioRender.com/gsenbm1

In summary, multidimensional lifestyle medicine holds high preventive and therapeutic potential in PD (Fig. 1). Although the evidence for substance use cessation is conflicting and most lifestyle factors (e.g., sleep quality) are prone to reverse causation, interventions on physical activity, nutrition, stress management, and social interaction are likely to reduce PD risk, delay PD onset, and slow PD progression. Given the long latency of PD even in at-risk populations, gold-standard class I prospective controlled interventional trials will not be feasible to prove the efficacy of lifestyle interventions on PD risk. Nevertheless, a growing number of well-conducted observational studies controlling for confounding factors along with biological plausibility is sufficient to recommend lifestyle modifications. Of note, recommendations derived from the current evidence for lifestyle medicine in PD are rather general, including moderate to high physical activity and a Mediterranean-like diet. Future studies will therefore need to develop and assess more disease-specific interventions to maximize beneficial effects and to strengthen evidence for true causal effects. In addition, the limited consideration of PD subcohorts might have obscured benefits in previous studies. Moreover, it will be highly interesting to examine multidimensional interventions assessing and targeting several domains of lifestyle medicine. This is particularly relevant, as a strong interaction between these domains is known. For example, sleep quality is affected by diet (Farrell et al. 2024), and physical activity, smoking, and diet interactively modulate the gut microbiome in PD (Heinzel et al. 2021). Importantly, integrative lifestyle adaptation to optimize several protective factors may have a superadditive effect on PD risk reduction (Kim et al. 2018). To achieve this aim, future prodromal or manifest patient cohorts can be better defined based on biomarkers including positivity in synuclein seed-amplification assays. Stratification by other markers such as microbial signatures will further allow an identification of subgroups particularly responsive to lifestyle interventions and the development of more individualized approaches. Such specific interventions further require a better mechanistic understanding of lifestyle interventions including potential neuroprotective effects.

### Limitations

The limitations of studies included in this review and the challenges to be addressed in the future are the missing consideration of phenotypic heterogeneity in PD, in particular the underrepresentation of late disease stages and cognitive impairement in PD patients. Moreover, studies frequently lack adequate “placebo” controls and describe very low numbers of enrolled patients with short-term interventions partially being due to limited financial support in contrast to pharmacological interventions, resulting in restrictions in study design (Ebersbach 2025). Importantly, many studies on the role of lifestyle and lifestyle-targeted interventions in PD are of correlational nature lacking sufficient evidence for causality. There is particular susceptibility for bias at many levels, including causation bias, recall bias, information bias, confounding bias, and selection bias towards patients more motivated to adhere to interventions compared to the general population. Thus, a major limitation of this review is the lack of a structured risk-of-bias assessment for the referenced studies. Moreover, the reliance on secondary sources (reviews, meta-analyses) can further propagate biases. Finally, this is a narrative, non-systematic review based on a single-database search.
